# Nationwide incidence of lateral malleolar fracture surgery across 6 European countries: has recent evidence changed clinical practice?

**DOI:** 10.2340/17453674.2025.44797

**Published:** 2025-10-10

**Authors:** Ville PONKILAINEN, Thomas IBOUNIG, Tim JONES, Aleksi REITO, Tom J CRIJNS, Michael WHITEHOUSE, Li FELLÄNDER-TSAI, Cyrill SUTER, Lasse RÄMÖ, Teppo L N JÄRVINEN

**Affiliations:** 1 Department of Orthopaedics and Traumatology, Tampere University Hospital, Wellbeing Services County of Pirkanmaa; 2 University of Tampere, Tampere; 3 Finnish Centre for Evidence-Based Orthopaedics (FICEBO), University of Helsinki;; 4 Department of Orthopaedics and Traumatology, Helsinki University Hospital, Finland; 5 Musculoskeletal Research Unit, Translational Health Sciences, Bristol Medical School, University of Bristol, Learning and Research Building, Level 1, Southmead Hospital, Bristol, UK; 6 Department of Clinical Science, Intervention and Technology, Division of Orthopaedics and Biotechnology Karolinska Institutet, Stockholm, Sweden; 7 Department of Orthopedic Surgery, Mayo Clinic, Phoenix, AZ, USA; 8 National Institute for Health Research Bristol Biomedical Research Centre, University Hospitals Bristol and Weston NHS Foundation Trust and University of Bristol, Bristol, UK

## Abstract

**Background and purpose:**

Increased use of weightbearing radiographs to assess ankle mortise stability have suggested that most lateral malleolar fractures with a congruent mortise on initial radiographs can successfully be treated nonoperatively. We aimed to evaluate trends in the surgical management of isolated lateral malleolus fractures across Austria, England, Finland, Germany, Sweden, and Switzerland from 2013 to 2022

**Methods:**

We performed a multi-register study to document the annual incidence of operative treatment for isolated lateral malleolus fractures through procedure codes across 6 European countries between 2013 and 2022. The annual incidence of operative treatment was calculated by dividing the total number of procedures per year by the year- and age-matched population based on publicly available demographics data.

**Results:**

Across the 6 studied European countries, the incidence of surgery for lateral malleolar fracture varied 6-fold between the country with highest (Germany) and lowest (England) incidences; Germany: 37 (95% confidence interval [CI] 37–38) per 10^5^; Switzerland: 34 (CI 32–35) per 10^5^, Austria: 27 (CI 26–28) per 10^5^, Finland: 17 (CI 16–18) per 10^5^, Sweden: 8 (CI 7–9) per 10^5^, and England: 6 (CI 6–7) per 10^5^ in 2021. Over the 10-year study period, the incidence of surgery for lateral malleolar fractures declined notably in Sweden (–29%), Finland (–26%), England (–20%), and Switzerland (–14%), but remained stable in Germany and Austria.

**Conclusion:**

The incidence of surgery for lateral malleolar fracture varied 6-fold across 6 studied European countries. Reductions of approximately 20–30% were observed in England, Sweden, and Finland (countries with the lowest baseline rates), while in Germany and Austria (countries with higher baseline rates), the incidence of surgery remained stable over the 10-year observation period.

The incidence of ankle fractures is reported to be around 100–150 per 100,000 in Western countries, with a marked increase occurring after the age of 40 [1-5]. Approximately 60% of all ankle fractures are isolated (unimalleolar) Weber B type lateral malleolus fractures [6,7]. For these fractures, the stability of the ankle mortise is of fundamental clinical importance as an “unstable” ankle mortise is widely regarded as an indication for surgery [8,9].

Traditionally, the stability of the ankle mortise in congruent Weber B fractures has been assessed by evaluating displacement on radiographs or through various stress-testing techniques performed under fluoroscopy [8,10-12]. However, these methods have faced criticism for their limited accuracy in differentiating truly unstable fractures—those that become occultly incongruent in a cast, and thus, require surgical stabilization—from stable fractures that remain congruent in a cast and can thus be successfully managed nonsurgically [13-15].

However, over the past 15 years, we have witnessed the emergence of a new approach for assessing stability of the ankle mortise in isolated fractures of the lateral malleolus: the use of weightbearing radiographs. At the initial encounter, a fractured ankle with a congruent mortise is placed in a cast, the patient is instructed to bear weight as tolerated, and then congruency is reassessed radiographically, typically 10 to 14 days after injury [16]. Prospective studies using the approach have reported the rate of subsequent incongruency requiring surgery to be as low as 1–10% [13,17-21]. Use of the weightbearing radiograph protocol has been increasingly endorsed in some regions to decide whether surgery is necessary [17,22]. Also, a randomized controlled trial conducted in the United Kingdom—the Ankle Injury Management (AIM) trial [23]—provided further evidence challenging the long-standing practice of routinely performing surgery for patients with ankle fractures deemed “unstable.”

We aimed to evaluate whether the incidence of operative treatment for isolated lateral malleolar fractures varies across Austria, England, Finland, Germany, Sweden, and Switzerland, and whether it has declined in response to emerging evidence.

## Methods

### Study design and setting

We conducted a multi-register study using ankle fracture data from Austria, England, Finland, Germany, Sweden, and Switzerland. These countries were selected because they represent relatively similar European populations, but differing slightly with their healthcare and reimbursement systems. In Finland, England, and Sweden, healthcare systems are predominantly financed through taxation, while in Austria, Germany, and Switzerland they rely mainly on insurance-based funding. Moreover, each maintains a comprehensive nationwide registry from which we could obtain surgical information. In all 6 countries, ankle fractures undergoing operative treatment (open reduction and internal fixation) were identified from hospital episode statistics (HES) from admitted patient care data. The study period spanned from 2013 and 2022 in all countries except for Sweden where there was data available through 2021. The coverage of the registers used from England, Finland, and Sweden is shown to be high, with over 95% of discharges accurately recorded and positive predictive values for common diagnoses ranging from 75% to 99% [24-26].

The study is reported according to STROBE guidelines.

### Population

Age was restricted to the adult population, with the age threshold defined by each register. Patients with diagnosis code “fracture of lateral malleolus” (S826) as their primary surgical diagnosis were included from each data source. Data for Austria, Germany, and Switzerland is drawn from mandatory hospital discharge registers, upon which inpatient-care reimbursement is based. Consequently, the registers in Austria, Germany, and Switzerland are believed to achieve nearly 100% coverage [27-29], although no validation studies have yet been published. The differences between the countries’ data sources and the procedure codes are outlined in the Table and further detailed in Supplementary Table 1.

**Table:** Differences in data across Austria, England, Finland, Germany, Sweden, and Switzerland with diagnosis code ICD-10 = S82.6: fracture of lateral malleolus (lateral malleolar fractures)

Country	Age limits	Register	Procedure codes	Time period
Austria	≥ 19 years	Hospital Discharge Register	NC041 Osteosynthese der distalen Fibula	01-01-2013 to 31-12-2022
England	≥ 16 years	Hospital Episode Statistics	Supplementary Table 2	01-04-2013 to 31-03-2023
Finland	≥ 18 years	National Hospital Discharge Register	NHJ10 Fracture surgery of ankle and foot	01-01-2013 to 31-12-2022
Germany	≥ 20 years	National Registry	Supplementary Table 2	01-01-2013 to 31-12-2022
Sweden	≥ 18 years	National Hospital Discharge Register	NHJ60 Osteosynthesis of fracture in the ankle or foot with plate and screws, lateral malleolus NHJ69 Osteosynthesis of ankle fracture with plate and screws	01-01-2013 to 31-12-2021
Switzerland	≥ 19 years	Medical Statistics of Hospitals database	Supplementary Table 2	01-01-2013 to 31-12-2022

### Outcome

We reported the annual incidence of lateral malleolar fracture surgeries (per 100,000 person-years) stratified by country during 2 periods, for Austria, England, Finland, Germany, and Switzerland (2013–2022), and Sweden (2013–2021),

### Statistics

Data from each register was cleaned into a similar tidy format and then merged, including the variables: country, year, age group, case count, reference population, and incidence. Following data merging, standard analytical techniques for aggregated data were used.

Annual mid-year populations, obtained for each country’s national statistics, were used for the calculations. Incidence rate ratios (IRRs) with 95% confidence intervals (CIs) were calculated using Poisson regression for each year, using the mean incidence of the first 3 complete data collection years (2013–2015) as the reference. The first 3 study years were selected to serve as a pragmatic baseline reference, allowing cross-country comparisons without anchoring the analysis to a single external event. The population, corresponding to each year and age group, was used as the offset in the model. Annual incidence rates were age-standardized using the 2013 European Standard Population, based on age distributions from 18–60 years and over 60 years, obtained from Eurostat [30]. Annual surgical incidence for lateral malleolar fractures was stratified into 2 age groups: age < 60 years vs age ≥ 60 years. All analyses were performed using R version 4.4.1 (R Foundation for Statistical Computing, Vienna, Austria). This manuscript was prepared in accordance with the STROBE checklist [31].

### Ethics, registration, data sharing plan, funding, and disclosures

Ethics approval was granted for Swedish data (ID 2022-04877 and 2020-04776). Approval was not required for other countries due to the retrospective and de-identified nature of the data. All procedures followed ethical standards in accordance with the Declaration of Helsinki. The data used in this study cannot be shared publicly due to restrictions imposed by Finnish data protection legislation.

This study was supported by the NIHR Biomedical Research Centre at University Hospitals Bristol and Weston NHS Foundation Trust and the University of Bristol. The views expressed are those of the authors and not necessarily those of the NIHR or the Department of Health and Social Care.

Each author certifies that he or she has no commercial associations (e.g., consultancies, stock ownership, equity interest, patent/licensing arrangements, etc.) that might pose a conflict of interest in connection with the submitted article. Complete disclosure of interest forms according to ICMJE are available on the article page, doi: 10.2340/17453674.2025.44797

## Results

**Figure 1:**
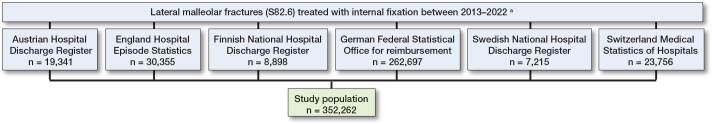
Flowchart of included patients.

During the study period, a total of 19,341 lateral malleolar fractures were treated with internal fixation in Austria, 30,355 in England, 8,898 in Finland, 262,697 in Germany, 7,215 in Sweden, and 23,756 in Switzerland, comprising a total population of 352,262 fractures between 2013 and 2022 (Figure 1).

**Figure 2:**
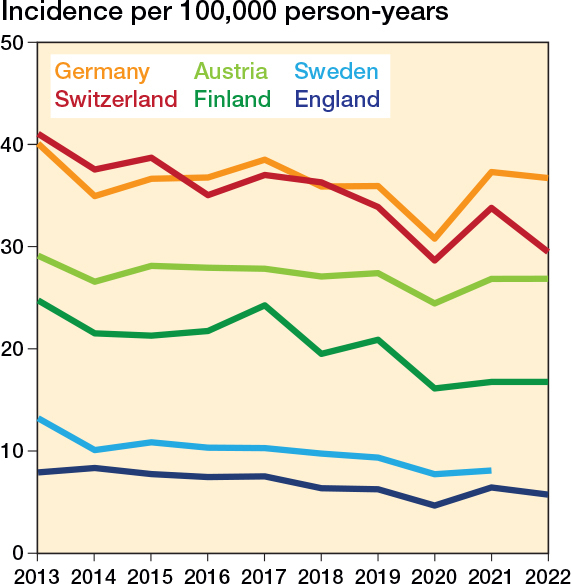
Incidence of lateral malleolar fracture surgeries in Austria, England, Finland, Germany, and Switzerland 2013–2022 and Sweden 2013–2021.

The incidence of surgery for lateral malleolar fractures varied markedly between countries throughout the study period (Figure 2). In 2021, the incidence per 100,000 person-years ranged from 37 (CI 37–38) in Germany—the highest observed—to 6 (CI 6–7) in England; the corresponding figures for the other countries were: Switzerland 34 (CI 32–35), Austria 27 (CI 26–28), Finland 17 (CI 16–18), Sweden 8 (CI 8–9).

**Figure 3:**
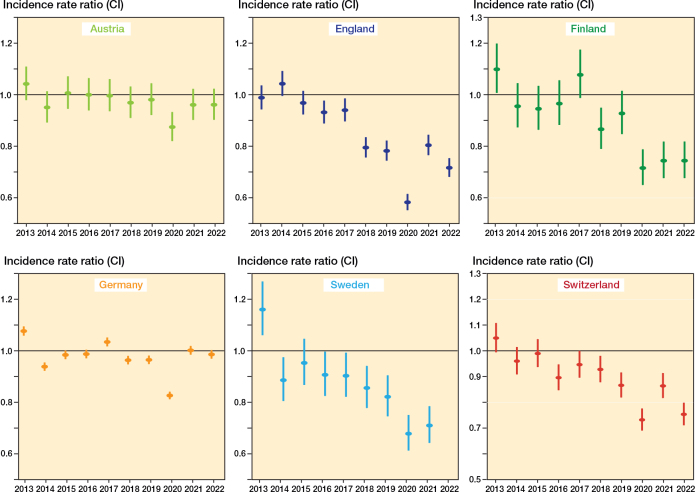
Incidence rate ratios (IRR) with 95% confidence intervals of internal fixations of lateral malleolus fractures over time, categorized by country. Mean incidence of years from 2013 to 2015 as the reference incidence.

Over the 10-year study period, we observed a notable decline in the incidence of surgery for lateral malleolar fractures in England, Finland, Sweden, and Switzerland (Figure 3, Supplementary Table 1). Between the baseline years (2013–2015) and 2021, the largest reduction was seen in Sweden (–29%, IRR 0.71 [CI 0.64–0.79]), followed by Finland (–26%, IRR 0.74 [CI 0.68–0.82]), England (–20%, IRR 0.80 [CI 0.77–0.85]), and Switzerland (–14%, IRR 0.86 [CI 0.82–0.91]). In contrast, the incidence remained stable in Austria (–4%, IRR 0.96 [CI 0.90–1.02]) and in Germany (0%, IRR 1.00 [CI 0.99–1.02]).

**Figure 4:**
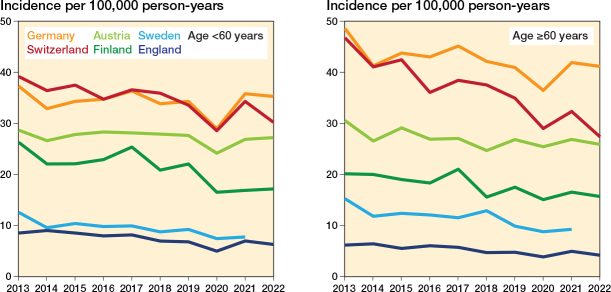
Annual incidence of lateral malleolar fracture surgeries in Austria, England, Finland, Germany, and Switzerland (2013–2022) and Sweden (2013–2021), shown separately for individuals < 60 and those ≥ 60 years of age

In addition to overall incidences, we also performed age-stratified analyses by dividing patients into 2 groups: < 60 years and ≥ 60 years old. This stratification was applied consistently across all countries and study years (Figure 4). In both age groups, Germany consistently showed the highest incidence of surgery, with England the lowest. Among patients under 60 years of age (Figure 4), the incidence in 2021 ranged from 36 (CI 35–36) per 100,000 in Germany to 7 (CI 7–7) in England, with intermediate rates in Switzerland, Austria, Finland, and Sweden. Among patients aged ≥ 60 years (Figure 4), the pattern was similar, with Germany again having the highest rate of 42 (CI 41–42) and England the lowest at 5 (CI 5–5) per 100,000 person-years.

**Figure 5:**
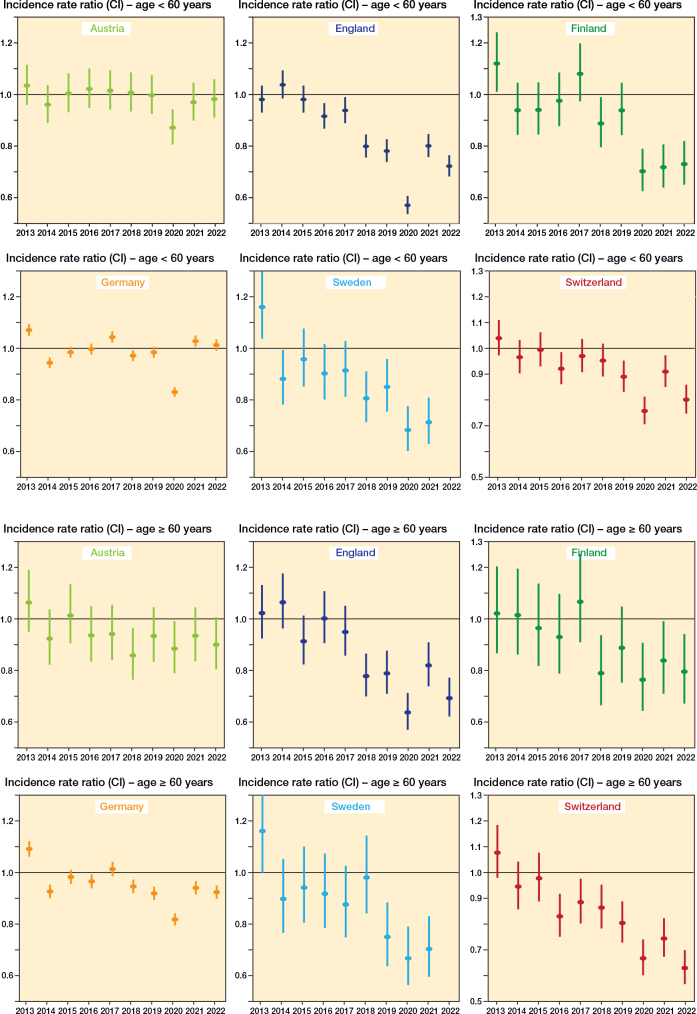
Incidence rate ratios (IRR) with 95% confidence intervals of internal fixations of lateral malleolus fractures over time, categorized by age group: < 60 years (upper 2 panels) and ≥ 60 years (lower 2 panels) and country. Mean incidence of years from 2013 to 2015 as the reference incidence.

Specifically, in patients under 60 years (Figure 5, Supplementary Table 1), the incidence declined in England, Finland, Sweden, and Switzerland, whereas rates in Austria and Germany showed little to no change. In patients aged 60 years or older (Figure 5, Supplementary Table 1), a decrease was observed in all countries, though of varying magnitude.

## Discussion

The management of lateral malleolar fractures is a subject of ongoing debate. Traditionally, lateral malleolar fractures with a congruent mortise on initial radiographs but of uncertain stability have undergone some form of stability assessment and—if deemed unstable—operative treatment. However, recent studies and guideline recommendations challenge this approach, advocating for placing such ankles in a cast and allowing weightbearing, and reassessing stability within 10–14 days using follow-up weightbearing radiographs without cast [18,20-22,32]. This emerging strategy could result in considerably reduced need for operative treatment of lateral malleolar fractures. Evidence suggests that when the weightbearing approach is used to assess ankle mortise stability, only 3–10% of patients ultimately require surgery [17,18,20]. If fully adopted, this shift in practice could transform the standard of care, prioritizing nonoperative management while minimizing unnecessary surgical interventions. We performed a multi-register study of 6 European countries to document 6-fold variation in the incidence of operative treatment for these fractures in the studied registries. In addition, while reductions of approximately 20–30% were observed in England, Sweden, and Finland (countries with the lowest baseline rates), the incidence of surgery remained stable over the 10-year observation period in Germany and Austria (countries with higher baseline rates).

The concept of practice variation, often referred to as “unwarranted variation,” was introduced and popularized in the 1970s by Wennberg and Gittelsohn [33]. It describes differences in the use of medical services that cannot be explained by variations in patient illness, preferences, or adherence to evidence-based guidelines. Instead, such variations are often driven by local medical culture, physician preferences, and systemic factors like resource availability and reimbursement system [34]. Regarding our finding of a 6-fold difference in the incidence of surgery for lateral malleolar fractures between the 6 studied European countries, it could be argued that some of the observed variation may stem from true differences in fracture incidence and severity, but it is highly unlikely that these factors alone could explain differences of the magnitude observed. Instead, the degree of variation observed likely reflects local medical practices and surgeon preferences [35-37]. Variations in reimbursement systems across the included countries may influence the financial incentives for surgeons, potentially affecting surgical rates. This factor could contribute to the comparatively higher rates observed in Germany, Switzerland, and Austria, countries with insurance-based healthcare; however, as no data on these system-level differences was available, this remains speculative.

While the documentation of practice variation is important in itself, another key objective of our study was to explore whether recent clinical evidence has influenced surgical practice in different countries. Specifically, we aimed to identify temporal trends that could suggest shifts in practice—whether associated with emerging evidence or other factors—alongside the observed between-country variation.

Our analysis revealed a decline in operative incidence over the study period in England, Finland, Sweden, and Switzerland, while rates remained stable or slightly increased in Germany and Austria. These trends were evident both in the overall population (Figures 1 and 2) and within age-stratified analyses (Figures 3 and 4), with reductions generally more pronounced among younger patients.

In England, the decline in operative treatment rates coincided with the publication of the BOAST guideline published in 2016 [22], and may reflect its early uptake in clinical practice. Conversely, despite the AIM trial [23] specifically addressing treatment in older adults (≥ 60 years), we did not observe a corresponding age-specific shift in incidence rates in England. This suggests that the AIM trial’s findings had a limited impact on routine clinical practice within its target population.

In Sweden and Finland, the observed decreases began after 2020 and may reflect a gradual shift influenced by the regional dissemination of evidence from the studies by Gregersen and Molund in Norway [17]. Notably, the decrease in the incidence of operative treatment in England, Sweden, and Finland began between 2017 and 2019, pre-dating the pandemic and associated national lockdowns. This further supports the conclusion that these changes reflect genuine shifts in clinical practice rather than temporary, pandemic-related disruptions.

In contrast, surgical rates in Germany and Austria showed little to no long-term change. Although a minor transient decrease was observed during the COVID-19 pandemic in both countries, rates returned to baseline thereafter. This suggests that any impact of the pandemic on fracture incidence and associated surgical volumes was temporary and did not alter established surgical practices.

Despite accumulating evidence supporting nonoperative management of stable lateral malleolar fractures, the observed reductions in operative treatment rates have remained modest, particularly and perhaps also paradoxically in the countries with higher baseline incidences (Germany and Austria). This highlights a broader challenge in surgical practice: the often slow and variable adoption of new evidence, particularly when it contradicts long-standing norms or entrenched clinical routines [37]. Factors contributing to this inertia include cognitive biases, the entrenchment of established norms, and clinicians’ confidence in traditional interventions [38].

Given the profound financial and health implications of medical overuse, the lessons learned from successful practice changes—such as the approximately 90% reduction in rates of arthroscopic partial meniscectomy and subacromial decompression in Finland [38,39]—could guide strategies for aligning ankle fracture management more closely with current evidence. Shifting clinical practice toward evidence-based approaches in ankle fracture management is essential, yet it remains a substantial challenge within the orthopedic community.

### Limitations

First, there may be coding inaccuracies inherent in any database research. Although differences in registry accuracy may influence absolute incidence rates between countries, we believe the large observed differences are unlikely to be explained solely by data quality or coding variation. Furthermore, possible differences in registry accuracy are less likely to affect within-country trends over time. To our knowledge, there were no major structural changes in healthcare, reimbursement systems, or registry operations in any of the included countries during the study period. We deliberately chose to use similar types of nationwide hospital registers across all countries to maximize comparability, even though this approach may have compromised the precision achievable with specialized fracture registries in individual countries. Second, changes in lifestyle and patient behavior, such as reduced physical fitness and exercise, may have contributed both to a decreased incidence of these injuries and to a lower suitability of individuals for surgery; however, these aspects could not be assessed in the present study due to data limitations. Third, we were unable to include data on nonoperative treatment of lateral malleolar fractures, as such cases are often treated in outpatient or primary care settings without systematic reporting to national registers. This limitation prevented us from analyzing overall treatment rates or treatment selection trends at the population level. Fourth, since the key evidence and guidelines were published between 2016 and 2021 [17,22,23], one could argue that the follow-up period may be too brief to fully capture longer-term practice changes. However, the use of weightbearing radiographs to assess mortise stability was introduced in 2010 [19], and supporting evidence has accumulated over the past decade. Given that we observed a notable decrease in operative treatment rates in several countries during our study period, we believe the timeframe is appropriate for detecting meaningful shifts in practice if they occurred. Finally, although registry-specific factors may influence recorded incidence rates, the consistency of observed trends within each country supports the robustness of our findings. Furthermore, substantial variation in procedure rates between European countries has been reported across multiple surgical fields, as shown in Eurostat statistics, which supports the external validity of our findings [40,41].

### Conclusions

We identified substantial variations in the incidence of operative treatment for lateral malleolar fractures across 6 European countries. The difference between Germany having the highest incidence and the lowest incidence, in England, was 6-fold. Additionally, we observed a roughly 20% to 30% decrease in the incidence of operative treatment for lateral malleolar fractures in England, Finland, Sweden, and Switzerland during the latter part of the study period. In contrast, the operative incidence in Germany and Austria has remained stable despite accumulating evidence supporting initial nonoperative treatment.

*In perspective,* these findings underscore the need for greater efforts to align clinical practices with the latest evidence-based recommendations to ensure consistent, effective, and economically sustainable care in each country.

### Supplementary data

Supplementary data and Tables S1–S2 are available as supplementary data on the article page, doi: 10.2340/17453674.2025.44797

## Notes

VP: conceptualization, methodology, formal analysis, investigation, and preparation of the original draft. TI, TJ, AR, TC, MW, LF-T, CS, LR, TJ: conceptualization, methodology, data acquisition, review and editing.

The authors sincerely thank Lauren Scott for her Stata code from Scott et al. (2020) [42], which was amended to provide relevant information for this study.

Handling co-editors: Cecilia Rogmark and Jonas Ranstam

*Acta* thanks Emilia Möller Rydberg for help with peer review of this manuscript.
